# Neovascular Glaucoma at King Khaled Eye Specialist Hospital – Etiologic Considerations

**DOI:** 10.4103/0974-9233.48860

**Published:** 2009

**Authors:** Hanan N. Al-Shamsi, David K. Dueker, Sawsan R. Nowilaty, Sami A. Al-Shahwan

**Affiliations:** 1 From the Department of Pediatric, King Khaled Eye Specialist Hospital, Riyadh, Kingdom of Saudi Arabia; 2 From the Department of Glaucoma, King Khaled Eye Specialist Hospital, Riyadh, Kingdom of Saudi Arabia; 3 From the Department of Vitreoretina, King Khaled Eye Specialist Hospital, Riyadh, Kingdom of Saudi Arabia

**Keywords:** Neovascular Glaucoma, Diabetic Retinopathy, Hypertension, Saudi Arabia

## Abstract

**Background:**

Neovascular glaucoma (NVG) is a severe form of secondary glaucoma caused by the growth of new vessels over the trabecular meshwork. The principal causes are associated with retinal ischemia. Ablative treatment of the retina can prevent, halt, and even reverse the growth of new vessels on the iris and angle. It is an essential part of the management in most cases.

**Aims:**

To determine the causes of NVG among Saudi patients, presented at the King Khaled Eye Specialist Hospital.

**Methods:**

A retrospective review of 337 Saudi patients with NVG was obtained. All cases were reviewed for the evidence and causes of the disease, and their basic demographic information. A subset of 100 diabetic patients with PDR was further studied in greater detail for clinical findings and treatment history.

**Results:**

The most common primary etiologic associations for NVG included diabetic retinopathy (DR) (56.06%), retinal venous obstruction (26.40%), and chronic retinal detachment (03.56%). A history of diabetes mellitus was reported in 65.04%, systemic arterial hypertension was noted in 61.00%, and evidence of renal impairment was documented in 22.00%. Vision was markedly reduced in most eyes with NVG (median: hand motion). The median best visual acuity in the fellow eye was 20/160. Among the 100 cases, with DR as a cause of NVG, 43 patients had bilateral neovascularization of the iris (NVI) and 72 had bilateral PDR. Sixty-one patients had no previous laser treatment before the diagnosis of NVG. Among these, who received treatment, the median number of total laser spots was 1,003.

**Conclusions:**

Diabetes is a major cause of NVG presented to this tertiary eye care center in the Kingdom of Saudi Arabia followed by retinal venous obstruction. Close monitoring and full pan-retinal photocoagulation (PRP) were absent in most of the diabetic cases. It is important to recognize that the “unaffected” fellow eye, particularly in diabetic patients, may require fairly urgent treatment as well.

Neovascular glaucoma (NVG) is classified as a secondary glaucoma which can complicate several ocular diseases characterized by retinal hypoxia. Its main feature is the proliferation of a fibrovascular membrane - composed of fragile, abnormal new vessels and myofibroblasts - which may cover the anterior chamber angle obstructing aqueous outflow.^1^ There have been a number of comprehensive reviews on NVG^2^–^5^ which have categorized the disorders underlying the development of this form of glaucoma. From these studies, there is a general agreement that approximately one-third of the cases of NVG are attributable to diabetic retinopathy, one-third to central retinal venous obstruction (CRVO), and one-third to diverse causes, with carotid artery occlusive disease being prominent. To determine if the causes of NVG were similar in our population, we gathered etiologic data and other relevant information on 337 patients with NVG who were managed at King Khaled Eye Specialist Hospital (KKESH) in Riyadh, from 1994 to 2004.

## MATERIAL AND METHODS

A search of KKESH medical records identified 785 cases of NVG during the period between 1994 and 2004. For the purpose of our retrospective study, we randomly selected the charts of 337 cases of NVG among Saudi patients for review, after the study had been approved by the hospital's Institutional Review Board. Included were only those cases in which iris neovascularization was noted to be present in association with increased intraocular pressure (i.e. greater than 22 mmHg). Basic demographic data were obtained for each patient, as well as the most likely cause of NVG as documented by the treating physician.

A subset of 100 diabetic patients with documented PDR was studied in more detail, with particular attention to treatment history prior to developing NVG. Excluded were diabetic patients with: 1) Undocumented status of the retina, 2) A possible alternative cause of NVG (e.g. CRVO, chronic rhegmatogenous retinal detachment), or 3) Inadequate records (essential data missing).

## RESULTS

The study included the records of 337 Saudi patients ranging in age from 3 to 92 years with a mean age of 62 years. Among this group, 190 (56.33%) were men and 147 (43.62%) were women. Of the 337 patients, 216 (64.09%) lived outside Riyadh and 121 (35.90%) were from Riyadh. The right eye was affected in (53.11%) and left eye was affected in (46.88%) of the cases. The best visual acuity (VA) recorded at the time of the diagnosis of NVG in the involved eye ranged from 20/30 to no light perception (NLP) with a median of hand motion (HM). The intraocular pressure (IOP) recorded at the time of diagnosis of NVG ranged from 23 mmHg to 79 mmHg with a mean of 40 mmHg. The most common etiologic factors found in this series were as follow (see Table 1):

**Table 1:** Etiological Factors Associated with NVG

	King Khaled Eye Specialist Hospital (1994-2004)		Wills Eye Hospital^5^ (1978-1981)	
		
Primary Contributing Cause of NVG	No. of Cases (% Total)	Mean Age in Years (Range)	No. of Cases (%Total)	Mean Age in Years (Range)
Diabetic retinopathy	189 (56.06)	62.4 (20-89)	67 (32.2)	54.7 (19-73)

Retinal venous obstruction	89 (26.40)	66.4 (20-95)	75 (36.1)	69.1 (19-89)
a. Central	82 (24.33)	66.4 (20-95)	67 (32.2)	69.3 (19-89)
b. Hemispheric	5 (1.48)	69.0 (55-84)	5 (2.4)	67.4 (61-81)
c. Branch	2 (0.50)	73.8 (55-95)	3 (1.5)	68.0 (60-76)

Unknown	27 (8.01)	71.5 (45-89)	9 (4.4)	55.2 (29-73)

Chronic RD	12 (3.56)	56.4 (15-89)	3 (1.5)	59.4 (29-77)

Carotid artery obstruction	6 (1.78)	64.6 (50-79)	27 (12.9)	64.2 (51-82)

Miscellaneous				
a. Retinoblastoma	9 (2.67)	06.7 (3-14)		
b. Uveitis	2 (0.50)	82.0 (23-84)	3 (1.5)	49 (41-57)
c. ROP	1 (0.29)			
d. Endophthalmitis	1 (0.29)			
e. Choroidal melanoma	1 (0.29)		1 (0.5)	

Total No. of Patients	337		208	

### Diabetic Retinopathy

PDR was the most common primary factor predisposing to the development of NVG and accounted for 56.06% (189/337) of the cases. Of these 189 patients, 100 cases were randomly selected to be studied in further detail.

Among these 100 cases of NVG secondary to PDR, 54 were men and 46 were women. A history of systemic arterial hypertension was noted in 35, and evidence of renal impairment was documented in 19 patients. The best VA in the involved eye at the time of the diagnosis of NVG ranged from 20/60 to NLP, with a median of (1/200). The median best VA in the fellow eye was also poor (20/400). The IOP of the involved eye at time of diagnosis ranged from 23 mmHg to 74 mmHg with a mean pressure of 43.3 mmHg (see Table 2). Twenty two patients had an increased IOP (>22 mmHg) in both eyes, 43 of the patients had iris neovascularization in both eyes while 72 of the 100 patients exhibited to have bilateral PDR.

**Table 2:** Characteristics of 100 Patients with PDR and NVG

	Involved Eye	Opposite Eye
Visual Acuity- range (median)	20/60 – NLP (1/200)	20/20 – NLP (20/400

Intra-ocular Pressure range (mean)	23 – 74 (43.3)	5– 62 (19)

Neovascularization of iris (%)	100	43

Proliferative Diabetic Retinopathy (%)	100	72

In this group of 100 patients with NVG secondary to PDR, 61 had had no laser treatment prior to the diagnosis of NVG. Of the 39 patients who did have prior laser treatment, 17 had this performed at KKESH and, therefore, a chart was available for review. In this group (n=17) the number of laser spots ranged from 48 (in one patient) to 4,072 spots, with a median of 1,003 spots. Ten of the 17 patients received less than 2000 spots of PRP. So, 61% of the patients with NVG from PDR had received no prior laser treatment of their retina; and, among those who did have some laser treatment, it appears that the majority had not received a full treatment - judging from cases where records were available.

### Retinal Vascular Occlusive Disease (RVOD)

RVOD was the second most common etiologic factor associated with NVG. Central retinal venous obstruction accounted for 24.33% (82/337) of the total cases, followed by hemicentral retinal vein occlusion in 1.48% (5/337) and branch retinal vein occlusion in 2 patients. Two patients had central retinal artery occlusion, one of which was also associated with CRVO.

### Chronic Retinal Detachment

Chronic retinal detachment accounted for 3.56% (12/337) of all cases of NVG. Two cases were due to Coat's disease.

### Carotid Artery Obstruction

Of the 337 patients of NVG, 6 (1.78%) were associated with clinical signs of ocular ischemic syndrome secondary to atherosclerotic carotid arterial obstruction. Confirmatory carotid doppler evidence of obstruction was only available in 2 cases.

### Miscellaneous Causes

Fourteen patients (4.15% of total) were included in this group (See Table 1). Nine patients had retinoblastoma and two had uveitic glaucoma. Choroidal melanoma, retinopathy of prematurity and endophthalmitis accounted for one case each.

### Unknown Aetiology

In 27 patients with NVG (8.01% of total) no underlying aetiology for the NVG was documented. Fifteen patients were diabetic and eight were hypertensive. The majority (70%) had NLP vision at presentation. Due to media opacities, the retina of sixteen patients could not be examined. The B-scan in these cases showed no posterior segment pathology.

## DISCUSSION

Our series agrees with the clinical study of the 100 patients reported by Hoskins^6^ and the 208 cases by Brown et al,^5^ in which diabetic retinopathy and central retinal vein obstruction were found to be the leading causes of NVG. Both are diseases that compromise the retinal vasculature, producing retinal ischemia, and the likely elaboration of angiogenic factors. In our series, diabetic retinopathy was the leading primary cause of NVG and diabetes was, overall, the most commonly associated primary systemic disease (65.04%). The diagnosis of diabetes in all our cases, however, was made only by review of the past medical history. The true incidence of diabetes might be higher if each patient was tested with a glucose tolerance test.

In the current study, we noted that the age at the time of development of NVG was lower in patients with diabetes (62.4 years) than in those with retinal venous obstruction (66.4 years), or in people with carotid artery obstruction (64.6 years). However, these age differences were not statistically significant. A similar observation was made by Madsen^4^ and Brown et al.^5^

The data in this study is in agreement with the study done by Brown et al,^5^ suggesting that the presence of bilateral NVG strongly indicates diabetic retinopathy as the primary underlying cause. This is expected since diabetic retinopathy is usually bilateral and relatively symmetrical as compared to retinal vascular occlusive disease. The incidence of DR in bilateral NVG was as high as 88% whereas NVG was bilateral in only 8% of cases of RVOD. Therefore it is important to recognize that the fellow eye, particularly in diabetic patients, may require fairly urgent treatment as well.

In addition, as it has been reported by Brown GC et al,^14^ that the association of central retinal artery obstruction (CRAO) with NVG is strongly suggestive of severe atherosclerotic carotid artery disease. Eyes with NVG, following CRAO, should be more appropriately characterized as having the ocular ischemic syndrome if a concomitant severe carotid artery obstruction is present. As none of our patients with NVG after CRAO underwent carotid angiography, it is very likely that cases of marked carotid atherosclerosis remained undetected. Some cases of atherosclerotic carotid obstruction were also most probably overlooked in the diabetic retinopathy and venous obstruction groups, because an obvious precipitating fundus abnormality was present and carotid artery angiography was therefore rarely performed.

Although our study did not address the prevalence of diabetes in Kingdom of Saudi Arabia, it demonstrates clearly that diabetes, followed by retinal vascular occlusive disease, is the major cause of NVG presenting to a tertiary care eye hospital. Diabetes mellitus (DM) continues to be one of the most common systemic diseases among the general population in KSA. Al Nozha et al^7^ assessed the prevalence of DM in Saudi Arabia using a community-based national epidemiological health survey of 16,917 Saudi adults enrolled over a 5-year period between 1995 and 2000. They reported that the overall prevalence of DM was 23.7%, and that diabetes mellitus was more prevalent among people living in urban areas (25.5%) compared to those in rural settings (19.5%), (p<0.00001). The study pointed out that despite the easy accessibility to healthcare facilities in KSA, 27.9% of diabetics were unaware of having the disease.

Judging by blood glucose records and patient reports in the medical history, the majority of our diabetic patients exhibited poor diabetic control. Although 60% of our patients were documented to have Type 2 DM, 63% of them were receiving insulin. In addition, a history of systemic arterial hypertension was found in 61% of our subjects, and 22% had some degree of renal impairment, both of which are known to exacerbate diabetic retinopathy. With the publication of the Diabetic Controls and Complications Trial (DCCT) results, there is now convincing evidence that near-normal glycemia is associated with delayed development and lesser severity of diabetic retinopathy.^8^

It is recognized that PRP can cause regression of new vessels in the anterior chamber in patients with DR.^9^ This observation is consistent with the well-documented efficacy of retinal ablation in preventing neovascular glaucoma as a complication of diabetic PDR and of CRVO and; although it is less thoroughly studied in other diseases causing NVG, laser retinal ablation is still considered effective in many of these as well. Among the 337 patients with NVG included in this study, we cannot predict, precisely, how many cases of NVG may have been prevented by early and adequate retinal ablation. But, based on the strong evidence for the ability of PRP to prevent and/or reverse anterior segment neovascularization, it is reasonable to project that a substantial number of these cases of NVG could have been avoided if the underlying disease process had been detected and treated effectively at an early stage.^10^

If early diagnosis and treatment could have had such positive benefits, it becomes interesting to consider why our study patients did not receive timely intervention. This question was addressed in a subsequent study performed by the authors between August 2004 and 2005 (unpublished data). Using an interview-based questionnaire given to 50 patients who had NVG secondary to PDR, we found serious deficiencies in the patient's knowledge about potential ocular complications of diabetes. One third of the patients responded that they were unaware that diabetes can affect their eyes, 68% had never received health education related to their diabetes, and 40% of patients had never had an eye exam before the onset of NVG.

A nationwide educational program could have profound benefits for preventing not only NVG, but also the wide variety of other ocular pathology associated with diabetes. The most important factor for preventing NVG in diabetics is the early detection of significant DR by regular ophthalmic examinations. The National Diabetes Advisory Board (USA) recommends that all newly diagnosed patients with Type II diabetes and all patients with Type I diabetes that has been present for longer than 5 years should have an annual examination by an ophthalmologist.^11^,^12^ *Diabetes 2000*, a program developed by the American Academy of Ophthalmology, specifically addresses the education of diabetic patients and medical colleagues on the importance of early diagnosis of diabetic eye disease, encouraging every ophthalmologist to teach his or her diabetic patients the importance of good glycemic control and regular eye examinations. The patients can then pass the information to their internist or family physicians. Such “reverse education” might prove very effective in alerting physicians in primary care to the benefits of recommending timely eye care for their diabetic patients.^13^

In summary, diabetes is a major cause of NVG at KKESH; and the occurrence of NVG, along with other diabetic eye problems, is likely to rise as the prevalence of diabetes increases in the Saudi population.

Our study shows that close monitoring and full PRP were absent in most of the diabetic cases. This highlights an opportunity to reduce serious complications of diabetes in the eye through early detection and treatment of diabetic eye disease. Education of both patients and primary care providers can help to address this important challenge. Increased awareness of diabetic eye disease and the benefits of timely treatment, coupled with convenient access to appropriate care, can decrease the incidence of this devastating form of glaucoma as well as other sight-threatening complications of diabetes.

## References

[1] Neovascular glaucoma, Martin Wand, Daniel Albert M Principles and Practice of Ophthalmology.

[2] Schulze RR (1967). Rubeosis iridis. Am J Ophthalmol.

[3] Gartner S, Henkind P (1978). Neovascularization of the iris (rubeosis iridis), Surv. Ophthalmol.

[4] Madsen PH (1971). Haemorrhagic glaucoma: comparative study in diabetic and nondiabetic patients. Br J Ophthalmol.

[5] Brown GC, Magargal LE, Schachat A (1984). Neovascular glaucoma: etiologic considerations. Ophthalmology.

[6] Hoskins HD (1974). Neovascular glaucoma: current concepts. Tr Am Acad Ophthalmol Otolaryngol.

[7] Al Nozha MM (2004). Diabetes mellitus in Saudi Arabia. Saudi Med Journal.

[8] Diabetes Control and Complications Research Group (1993). The effect of intensive treatment of diabetes on the development and progression of long-term complications in insulin-dependent diabetes mellitus. N Eng J Med.

[9] Jacobson DR, Murphy RP, Rosenthal AR (1979). The treatment of angle neovascularization with panretinal photocoagulation. Ophthalmology.

[10] Wand M, Dueker DK, Aiello LM (1978). Effects of panretinal photocoagulation on Rubeosis iridis, angle neovascularization, and neovascular glaucoma. Am J Ophthalmol.

[11] (1984). National Diabetes Advisory Board: The prevention and treatment of five complications of diabetes: A guide for primary care physicians. Metabolism.

[12] Nathan DM, Singer DE, Godine JE (1986). Retinopathy in older type II diabetics: association with glucose control. Diabetes.

[13] Legorretal AP, Hasan MM, Peters AL (1997). An intervention for enhancing compliance with screening recommendations for diabetic retinopathy: a bicoastal experience. Diabetes Care.

[14] Brown GC, Magargal LE, Simeone FA (1982). Arterial obstruction and ocular neovascularization. Ophthalmology.

